# Control of rural house infestation by *Triatoma infestans* in the Bolivian Chaco using a microencapsulated insecticide formulation

**DOI:** 10.1186/s13071-015-0762-0

**Published:** 2015-05-01

**Authors:** David Eladio Gorla, Roberto Vargas Ortiz, Silvia Susana Catalá

**Affiliations:** Centro Regional de Investigaciones Científicas y Transferencia Tecnológica. CRILAR-CONICET, 5201 Anillaco, La Rioja Argentina; Programa Chagas, Servicio Departamental de Salud, Santa Cruz de la Sierra, Bolivia

**Keywords:** Chagas disease, *Triatoma infestans*, Vector control, Chaco region, Microencapsulated formulation

## Abstract

**Background:**

*Triatoma infestans*, the main vector of *Trypanosoma cruzi* (causative agent of Chagas disease) has been successfully eliminated over much of its original geographic distribution over the southern cone countries of South America. However, populations of the species are still infesting houses of rural communities of the Gran Chaco region of Argentina and Bolivia. This study reports for the first time a large-scale effect of a vector control intervention using a microencapsulated formulation of organophosphates and insect growth regulator on house infestation by *T. infestans*, in the southwestern region of Santa Cruz de la Sierra Department, within the Bolivian chaco.

**Methods:**

The vector control intervention included the treatment and entomological evaluation of 1626 individually coded and georeferenced houses with the microencapsulated formulation. House infestation by *T. infestans* was evaluated by active searches with fixed capture effort carried out before and after two, 16 and 32 months of the treatment application.

**Results:**

House infestation prevalence was 30.5% before the intervention, spatially aggregated in two clusters of 38 and 25 localities that showed 41% and 38% house infestation by *T. infestans*. Infestation prevalence was reduced to 2.4% two months after the intervention and remained very low (1.7%) until the end of the study after 32 months of the control intervention, without any other additional vector control intervention.

**Conclusions:**

The obtained results show an important long lasting effect on house protection against triatomine infestation in a region of known pyrethroid resistant populations of *T. infestans*, as the result of the slow release of the active ingredients, protected by the formulation microcapsule.

## Background

Chagas disease, produced by the infection of *Trypanosoma cruzi*, is one of the main endemic diseases of Latin American countries, affecting about seven million people [1]. *T. cruzi* is transmitted to mammals by haematophagous insect vectors of the Triatominae subfamily (Reduviidae). It is considered that all Triatominae species are able to transmit the parasite, although less than 10 of the species have epidemiological importance, because of their association with humans. More than 90% of the Triatominae species occur in the Americas, with a small group occurring in Asia (China, India and northern Australia) [2]. *T. cruzi* is mainly transmitted by the triatomine vectors in the Americas, the transmission route is via their faeces eliminated after a blood meal onto the human body or through the consumption of food contaminated by insect faeces. *T. cruzi* can also be transmitted in the absence of insect vectors by the congenital route, through uncontrolled blood transfusions and organ transplants, or laboratory accidents with contaminated material. Initially restricted to the Americas, Chagas disease is now globally distributed because of human migration, especially in the USA (where about 300,000 people are infected) and Europe (where nearly 90,000 people are affected, especially located in Spain) [3,4].

Vector control programmes and blood bank control, coupled with a reduction of the rural population that migrated to urban environments and a generalized improvement in the living standard of rural communities (with exceptions), has successfully reduced the burden of the Chagas disease in Latin American countries. Wide areas of South and Central America are now certified by the Pan American Health Organization (PAHO) as having interrupted the transmission of *T. cruzi* by allocthonous triatomine vectors and by blood transfusion, within the framework of the continental initiatives for the control of Chagas disease. The Southern Cone was the first of the initiatives (INCOSUR) that started in 1991 and coordinated efforts to interrupt *T. cruzi* transmission through blood transfusion and by *Triatoma infestans*, the species that once accounted for more than 50% of the Chagas disease vectorial cases in America. Bolivia was the last of the Southern Cone countries to create a national programme to control Chagas disease. The systematic effort that started towards 2000 resulted in a strong reduction of the new infections, especially in the Altiplano region, where PAHO certified the interruption of the vectorial transmission of *T. cruzi* by *T. infestans* in the La Paz Department [5].

Although very successful in the Southern Cone of Latin America, the control of *T. infestans* is not complete in the Gran Chaco region (that covers western Paraguay, northwestern Argentina and eastern Bolivia), where *T. cruzi* is still actively transmitted in hot spot areas with high prevalence of house infestation and high abundance of *T. infestans* populations. A number of concurrent factors were mentioned as preventing the successful control of *T. infestans* populations infesting houses of rural communities of the Gran Chaco. Among these factors, the unsustained systematic vector control interventions derived from instabilities of health policies, the very low efficacy of suspension concentrate pyrethroid formulation to eliminate *T. infestans* populations infesting peridomestic structures [6] and the occurrence of pyrethroid resistant populations of *T. infestans* were mentioned [7-9].

After the recognition of low efficacy of the suspension concentrate formulations of pyrethroids to control *T. infestans* populations in the Gran Chaco, alternatives were analysed after studies were carried out under laboratory and/or field conditions. These alternatives included the study of active ingredients different to pyrethroids and/or alternatives to the traditional suspension concentrate formulation, as imidachloprid [10], and cypermethrin for dog collars [11,12], fipronil as a ‘spot on’ [13,14], and cypermethrin as a ‘pour on’ formulation [15-17]. Habitat modification and biological control have also been studied as reported for the modification of peridomestic structures [18], and biological control agents (oophagous parasitoids [19], fungi [20], and virus [21]). Among the studied alternatives, the microencapsulated organophosphate formulations were the only one evaluated under field conditions and not only at the experimental scale but using big sample size interventions; the formulation is prepared as a base paint that can be combined with colour to give the house a nice appearance [22].

The objective of the study is to report on the effect of a microencapsulated formulation of organophosphate insecticides on the infestation by *T. infestans* in rural houses of the Bolivian Chaco. Although the treatment of houses was not specifically planned for the present objective, the existing situation was used to design the analysis of the results as an observational study based on the time since the houses received the treatment, to compare the house infestation by *T. infestans* before and after the intervention.

## Methods

### Study area

**Figure 1:**
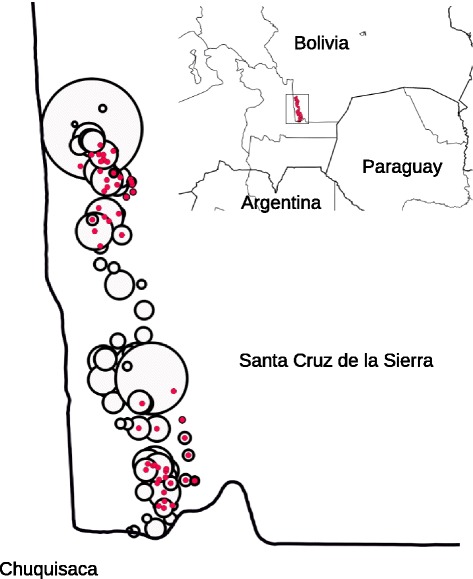
**Location of the 96 localities (white circles) of the study area.** Radius of circles are proportional to the number of houses in each locality. Red circles indicate localities within the north and south cluster of high house infestation by *Triatoma infestans*. Inset shows the study area location, south of Santa Cruz de la Sierra (Bolivia).

The vector control intervention included houses distributed in 96 localities of the municipalities of Cuevo and Lagunillas (Santa Cruz Department, Bolivia) (Figure 1). The treatment of houses was carried out two weeks after the following base line study (see below). A total of 1626 houses were treated, out of the 2197 houses in the region. Houses left untreated were either good quality ones in bigger towns or closed ones. The geographic coordinate of each house was recorded with handheld GPS receivers and received an individual code number that was used to carry out the followup along the study period.

The intervention was part of a project supported by the Generalitat Valenciana (Spain) that aimed at improving the living conditions of the rural communities, and was based on the application of a microencapsulated organophosphate formulation (Inesfly 5A IGR), containing diazinon, chlorpyriphos and the insect growth regulator pyriproxyfen (details of the formulation in [23], included in microcapsules that allow the slow liberation of the active ingredients from the treated surfaces.

The microencapsulated formulation was applied using a Jacto manual pump (HD-400 model, with nozzle JD-12P) to the internal and external walls, roof and all peridomestic structures (especially goat corrals, chicken coops and grain and tool deposits).

### Study design

The study included an entomological evaluation of the house infestation by *T. infestans* before intervention (base line survey) and another three entomological evaluations two, sixteen and thirty two months after the vector control intervention. Standard post-intervention timing for the evaluation of a pyrethroid insecticide against triatomines is one, six, and 12 months [24]. Because the microencapsulated formulation application took longer than the usual time needed to apply suspension concentrate formulations, the first entomological evaluation was carried out on average two months after the intervention. Knowing the longer lasting effect of the microencapsulated formulation under laboratory conditions [23], the posterior entomological evaluations were carried out 16 and 32 months after the intervention.

The entomological evaluations (either before or after the intervention) were carried out by agents of the Santa Cruz de la Sierra health authority (SEDES Santa Cruz), and consisted of timed active searches by a two-person team that made a 15-minute search within the house and another 15-minute search on the peridomestic structures, an adopted standard by the Pan American Health Organization for all Subregional Initiatives against vectors of *Trypanosoma cruzi. T. infestans* specimens were categorized as nymphs and adults, and identified by collection site (roof or walls in the intradomicile, and goat corral, chicken coop and grain and tools deposit in the peridomicile). House infestation prevalence was categorized as intradomestic (ID) or peridomestic (PD) and calculated as number of houses with *T. infestans* divided by the number of evaluated houses in the ID or PD. Insect density was calculated as number of collected insects by evaluated house or by infested house.

Base line data were collected through an entomological evaluation of *T. infestans* house infestation in 1712 houses, between November 2006 and May 2007. Of the 1712 evaluated houses, 1626 were treated with the microencapsulated formulation. The previous vector control intervention with insecticide (alfacypermethrin) in the study area, prior to the baseline survey was carried out in 2003.

Following the application of the microencapsulated formulation, a first entomological evaluation was carried out after two months (between March and July 2007). A second entomological evaluation was carried out 16 months after the treatment (between May and July 2008), and the third entomological evaluation (data available only for Lagunillas municipality) was carried out 32 months after the intervention, between May and October 2009. Table 1 synthesizes the number of treated houses and houses evaluated on each occasion during the studied period. The number of houses evaluated varied on each occasion, as owners were not always present and/or were not interested in participating in the project. House infestation prevalence (either ID or PD) was calculated as the number of infested houses divided by the number of houses evaluated on each occasion. Insect density is reported as an average per evaluated house and average per infested house.

**Table 1:** **House infestation by** ***T. infestans*** **before and after the intervention with the microencapsulated formulation**

	**No of houses**		**Infested houses**		**Collected insects**		**OR (CI 95%)**
**Months after intervention**	**Evaluated (infested)**	**Painted (infested)**	**ID**	**PD**	**ID**	**PD**	
0 (BL)	1712 (522)	1626 (502)	307	297	1459	2345	
2 (E1)	1947 (52)	1529 (46)	13	39	83	297	0.071 (0.052; 0.096)
16 (E2)	2096 (49)	1572 (32)	24	49	77	195	0.047 (0.033; 0.068)
32 (E3)	1064 (19)	1012 (18)	8	11	30	51	0.039 (0.024; 0.063)

ID and PD: number of evaluated and infested houses in intradomestic and peridomestic structures. BL is the entomological evaluation of houses before the microencapsulated application (baseline); E1, E2 and E3 are entomological evaluations after 2, 12 and 32 months after the microencapsulated application. Odds ratio (OR) and 95% confidence interval (CI95%) contrasts infestation prevalence at BL, with entomological evaluations after the microencapsulated application (E1, E2, E3).

### Data analysis

Comparison of house infestation prevalence was carried out calculating odds ratios. Comparison of *T. infestans* density among sampling dates was carried out through the estimation of a generalized linear model (glm function) with a Poisson link and the multiple comparison for glm (glht function) of the R package [25]. The detection of the spatial aggregation (clusters) of house infestation was based on each house coordinate and carried out using the ScanStat statistics [26], as described by [27]. Each cluster is defined as a group of localities that shows a significatively higher infestation than the average infestation prevalence in the area.

## Results

Houses were highly dispersed, with 62.9% of the localities having 60 or fewer houses. Of the total number of 2197 houses existing in the region, 36.2% were located in three localities having between 140 and 435 houses. Smaller villages had higher treatment coverage; 87% of houses were treated in villages with less than 100 houses, whereas 70% of them were treated in villages with more than 100 houses.

The base line entomological evaluation (before the intervention) showed that 17.9% (out of a total of 1712 evaluated houses) had intradomestic infestation, 17.3% had peridomestic infestation, and 4.8% had intra and peridomestic infestation, making an overall infestation of either intra and/or peridomestic infestation of 30.5%. The infestation was widespread, showing a dispersion index of 68.8% (66 out of 96 localities). The spatial analysis of house infestation showed there was significant aggregation of infested houses in two clusters. There was one cluster north of the study area with 38 localities, this included 451 houses, had 41.0% infestation, and there was another cluster south of the study area with 25 localities and 375 houses that had 38.4% infestation compared with 11.8% infestation outside the clusters, in 1221 houses of 33 localities (Figure 1).

Before the house treatment, average abundance (ID and PD) was 2.2 insects per evaluated house. It was significantly higher in chicken coops (5.9 insects/positive site) than in other sites (P < 0.05) and showed no difference between high infestation cluster localities and outside the cluster (P > 0.05) (Table 2).

**Table 2:** ***Triatoma infestans*** **density per infested house (D1) and per evaluated house (D2) in intradomestic and peridomestic sites, within (C) and outside (NC) of high infestation localities cluster (identified at the baseline entomological evaluation)**

			**Intradomestic**			**Peridomestic**		
			**Wall**	**Roof**	**Deposit**	**Wall**	**Chicken coops**	**Corrals**
LB	C	D1	4.83 (5.69; 95)	3.25 (3.86; 4)	3.78 (3.3; 18)	5.04 (5.52; 24)	10.16 (16.69; 61)	7.4 (7.83; 5)
		D2	0.72 (2.78; 638)	0.02 (0.37; 638)	0.11 (0.83; 638)	0.19 (1.42; 638)	0.97 (5.93; 638)	0.06 (0.9; 638)
	NC	D1	4.43 (3.99; 155)	3.33 (2.52; 3)	4.34 (4.09; 35)	6.33 (6.12; 75)	9.19 (10.45; 123)	1.88 (1.22; 17)
		D2	0.59 (216; 1160)	0.01 (0.2; 1160)	0.13 (1.02; 1160)	0.41 (2.2; 1160)	0.97 (4.42; 1160)	0.03 (0.27; 1160)
E1	C	D1	13 (13.75; 3)	0	22 (; 1)	7 (; 1)	6.64 (6.42; 11)	0
		D2	0.06 (1.16; 654)	0	0.03 (0.86; 654)	0.01 (0.27; 654)	0.11 (1.17; 654)	0
	NC	D1	2.57 (1.51; 7)	0	2.5 (; 2)	0	4.65 (2.65; 24)	0
		D2	0.01 (0.22; 1293)	0	0.003 (0.09; 1293)	0.01 (0.35; 1293)	0.08 (0.78; 1293)	0
E2	C	D1	2.00 (0.63; 6)	0	2 (; 1)	8 (; 1)	2.4 (2.15; 5)	0
		D2	0.02 (0.2; 667)	0	0.003 (0.08; 667)	0.01 (0.31; 667)	0.02 (0.27; 667)	0
	NC	D1	3.81 (5.47; 16)	0	0	3.75 (0.96; 4)	11.07 (16.37; 14)	1 (; 2)
		D2	0.04 (0.67; 1429)	0	0	0.01 (0.20; 1429)	0.11 (1.84; 1429)	0.001 (0.06; 1429)

Entomological evaluations: base line (LB), 2 months (E1) and 12 months (E2) after the application od the microencapsulated formulation. D1 figures represents overall insect density average per. infested house (standard deviation; number of infested houses); D2 figures represents density. average per evaluated house (standard deviation; number of evaluated houses). Standard deviation is calculated for n>2.

Two months after the microencapsulated formulation application, the aggregation of house infestation found in the base line study disappeared, i.e. no significant spatial cluster was identified. Intradomestic infestation was found in 13 houses, whereas peridomestic infestation was found in 39 (0.7% and 2.0% house infestation prevalence, respectively) (Table 1). Intradomestic infestation was found mainly in room walls (10 cases out of 13) and chicken coops (24 cases out of 39). Average intradomestic density was 6.4 insects per infested house and average peridomestic density was 5.3 per infested house. Overall insect density average (intra and peridomestic), considering the 1947 evaluated houses was 0.2 insects per house, a 11.4-fold reduction compared with the base line study (Table 2).

The entomological evaluation carried out sixteen months after the microencapsulated formulation application, showed that a group of 49 houses (2.3%) were infested in the intra and/or peridomestic structures (out of 2096 evaluated) and a significant (P = 0.04) small aggregation of high infestation appeared south of the high infestation aggregation detected during the base line study. The aggregate included eight infested houses within a group of nine localities that comprised 122 houses (6.6% intradomestic infestation within the cluster, compared with and infestation of 0.8% outside it) (Table 1). Average intradomestic density was 1.3 and 0.03 insects per infested house within and outside the high infestation aggregation, respectively. The most frequent sites of insect capture were chicken coops and room walls (Table 2).

In the entomological evaluation carried out thirty-two months after the microencapsulated formulation application, house infestation prevalence was 1.78% intra and/or peridomestic infestation (Table 1). Average density was 3.7 and 4.6 insects per infested house in the intra and peridomestic structures. Overall insect density was very low: 0.03 and 0.05 in the intra and peridomestic structures, representing a 27 and 11-fold reduction, respectively, compared with the baseline study. The most frequent sites of insect capture were chicken coops and room walls (Table 2).

Overall insect density (per evaluated house) decreased significantly (P < 0.001) after the microencapsulated application, maintaining very low values throughout the study period. Insect density per infested house maintained similar values along the study period (P > 0.05).

## Discussion

Vector control programmes in Latin American countries have successfully interrupted the vectorial transmission of *T. cruzi* in several areas of South and Central America. Control programmes of Chagas disease vectors started in the 1950’s and 1960’s as well structured institutions in Argentina, Brasil and Venezuela, but appeared later in other countries, like Bolivia. After some decades of underfunded and irregular activities, the National Programme of Chagas Disease in Bolivia started by the early 2000s a new institutional cycle of systematic activities that showed the most important impact over the disease [28]. Important advances on the vector control interventions in the Andean Valleys were obtained, although the region of the Bolivian Chaco still shows rural communities with a high proportion of houses infested by *T. infestans*, similar to the situation occurring in a number of areas in the Gran Chaco region of Argentina. Several reasons have been invoked to account for the failure of vector control interventions in the Gran Chaco region of Argentina, Bolivia and (at a minor extent) Paraguay. Among them, political instabilities and fund shortage, low efficacy of pyrethroid insecticides and pyrethroid resistance [6,29].

One of the reasons for the low efficacy of the vector control interventions in the Bolivian Chaco became clear after 2001, when the existence of *T. infestans* populations resistant to pyrethroids was identified in northern Argentina and then in several areas within the Bolivian Chaco [29]. Resistance to pyrethroid insecticides of several studied *T. infestans* populations is high or very high [30], and resistance to other active ingredients (e.g. fipronil) never used for vector control in the area has also been found. This situation is attributed to the high genetic variability of the *T. infestans* populations occurring in the area that is associated with the centre of origin from which the species expanded to occupy most of the southern cone of South America [31-33]. Two additional reasons (and probably more extended than insecticide resistance) were identified as causing the low efficacy of pyrethroid insecticides for the control of triatomines in the Gran Chaco region. One is the rapid degradation of the active ingredient molecules caused by UV light, giving a residual activity of no more than two weeks [6]. The other is the survival of the residual triatomine population in the complex peridomestic structures usually present in the rural communities of the Gran Chaco [27].

This study is the first carried out in Bolivia using individualized coding for individual houses and the first to report the effect of a vector control intervention using a microencapsulated formulation of organophosphates over the infestation by *Triatoma infestans*, the main vector of *Trypanosoma cruzi* in the southern cone countries of South America. As found in other areas [27], house infestation prevalence showed significant spatial aggregation before the control intervention in the base line entomological evaluation of the present study. House infestation prevalence aggregation is usually promoted (among other factors) by the house quality, and this might be the aggregation cause in this case. The spatial aggregation of house infestation was not detected on the three entomological evaluations after the control intervention.

Although not tested directly, recent reports suggest that some *T. infestans* populations occurring in the study area are resistant to pyrethroids [8]. Even under this difficult scenario that pose difficult vector control situations in other areas where suspension concentrate formulations of pyrethroids are used, the intervention using the microencapsulated formulation maintained a very good control level, keeping the house infestation below 1.8% after 32 months of the house treatment, without the addition of any other vector control intervention. The microencapsulated formulation produces a longer effect on the *T. infestans* population mortality than that observed after the application of the traditional pyrethroid-based suspension concentrate formulation, that in the absence of additional control measures allow the recovery of the original *T. infestans* population abundance between one-three years after the intervention [34,35]. The contrast between formulations is attributed to the mechanism of slow release from the microencapsulated formulation [23] and to the protection that the microcapsules offer on the active ingredients, that normally is rapidly degraded because of the effect of environmental factors (UV, surface porosity, high temperatures) as shown by [6]. A similar long lasting effect of the organophosphate microencapsulated formulation on a nearby region of Santa Cruz and Tarija Departments (Bolivia) was reported in a study by [36], where they showed 22% mortality of locally collected fifth instar nymphs and adult *T. infestans* after wall bioassays on houses treated 34 months before. The same microencapsulated formulation, either based on organophosphate or pyrethroid active ingredients showed successful control on a number of insect pests, like pyrethroid-resistant mosquitoes [37,38], a palm pest curculionid [39], ants [40] and tsetse flies in Cote d’Ivoire [41].

Findings of this study should be considered within the limitations imposed by its own design. As there is no control group, we can not attribute the effect of the microencapsulated formulation as the unique cause for the elimination of *T. infestans* house infestation, as the outcome might be combined with effects derived from a behavioural change of the rural communities that kept rooms clean and tidy, or because vectors were easier to detect (and kill) against a recently painted wall. However, all past published evidence suggest that the microencapsulated formulation is at least and by far the most important driving cause of *T. infestans* reduction in the intervened communities.

The cost of the vector control intervention using the microencapsulated formulation is about 120 US dollars, for an average house with 300–320 square meters. Considering the longer lasting effect, the figure is below the cost of the standard intervention using pyrethroid insecticides, that ranges between 25 and 65 U$ per house [42,43] respectively. Inflation adjusted dollars to a 1999 common base give 85.8, 35.2, 67.5 (calculated through https://cps.ipums.org/cps/cpi99.shtml) for the microencapsulated formulation, lower and upper extremes of pyrethroid-based intervention, respectively.

For the case of domestic triatomine control, besides the long lasting effect, the microencapsulated formulation has an additional advantage over other formulations, as it can be applied as a wall paint with colour selected by the house owner. Although perceived as a minor element at the beginning of the vector control intervention, it came out as an important factor for the subsequent effect on triatomine recolonization. As house owners perceived the embellished house as a nicer place to live in, they were keener on maintaining house cleanliness and order, thus decreasing the chance of a reinfestation by triatomines [22].

## Conclusions

This study, carried out in the Bolivian Chaco (southwest Santa Cruz de la Sierra Department), shows that a microencapsulated formulation carrying organophosphates and insect growth regulator as active ingredients is cost-effective for the reduction of house infestation prevalence by *T. infestans* and the maintenance of the low prevalence figures (<2% after 32 months of the vector intervention) for a longer period than equivalent vector control interventions based on suspension concentrate formulations applied with spraying pumps.
